# Cytokines associated with Burkitt’s lymphoma in western Kenya

**DOI:** 10.1186/s13104-017-2841-0

**Published:** 2017-10-27

**Authors:** Isaac Ndede, Simeon K. Mining, Kirtika Patel, Fredrick M. Wanjala, David Chumba, Constance Tenge

**Affiliations:** 1 Department of Immunology, Moi University School of Medicine, P. O. Box 4606, Eldoret, 30100 Kenya; 2 Department of Biological Sciences, University of Eldoret, Eldoret, Kenya; 3 Department of Histopathology and Cytology, Moi University School of Medicine, Eldoret, Kenya; 4 Department of Child Health and Paediatrics, Moi University School of Medicine, Eldoret, Kenya

## Abstract

**Objective:**

Burkitt’s lymphoma (BL) is a common aggressive non-Hodgkin’s lymphoma in East and Central Africa among children. Persistent infections with Epstein Barr virus or *Plasmodium falciparum* are associated with immune hyperstimulation. It is hypothesised that inadvertent cytokine responses to infections indirectly or directly influence B cell neoplastic transformation through c-myelocytomatosis (*c*-*myc*) gene translocation. We sought to describe cytokines in children and adolescents with BL. Participants were recruited from western Kenya with parental consent, diagnosis confirmed using histology and consensus panel of immunohistochemistry antibodies. T helper1/2/17A and transforming growth factor-β1 (TGF-β1) cytokines were estimated using cytometric bead array in plasma. Complete blood counts (CBC) were determined by Beckman Coulter^®^.

**Results:**

Out of 104 enrolled participants, 32% were confirmed BL and 68% grouped as non-BL. Mean (pg/ml) levels of cytokines in BL and non-BL were: interleukin (IL)-6 100.3 and 39.4 p = 0.152; IL-10 11.5 and 12.5 p = 0.363; IL-17A 17.8 and 64.9 p = 0.094 respectively. Expressions of interferon-γ, IL-2 and tumour necrosis factor-α were low and TGF-β1 undetectable in both groups. Mean CBC differed between the two groups before and after chemotherapy, WBC being significantly so. Interleukin-6, IL-17A and IL-10 responses to infections in the study area may be associated with pathogenesis and be potential therapeutic targets.

## Introduction

Non-Hodgkin’s lymphomas (NHL) are the most common lymphomas in East and Central Africa [1], Burkitt’s lymphoma (BL) is a type of NHL, most frequent in children in this region [2], much more than in West or North Africa [3]. Three BL variants namely: sporadic (sBL), endemic (eBL), and immunodeficiency related BL are recognised worldwide. The endemic type accounts for 50–75% of cancers in children in East and Central Africa with an incidence rate of 5–10 cases per 100,000 children [4]. In Kenya, the incidence rate of BL is estimated to be 0.61 in 100,000 children [5].

Endemic BL epidemiology overlaps ecological zones associated with endemic malaria, Epstein-Barr virus (EBV) and other infectious diseases in the region [6]. Within a zone, BL incidence rate follows malaria transmission intensity [7]. Chronic infectious agents interact with the host immune system and may indirectly or directly initiate B cell proliferation. Prolonged immune-infectious agent interactions may lead to immune hyperstimulation and trigger genetic aberrations including *Ig/c*-*myc* gene translocations found in many lymphomas including BL. The mechanism by which persistent infections cause neoplastic transformation remains obscure. Inflammatory and/or anti-inflammatory cytokines produced in response to persistent infections including malaria have been linked to genetic changes which may lead to malignant transformations in some B cell tumours [8]. Some epidemiological data have implied that inflammatory and/or anti-inflammatory cytokines in response to persistent infections markedly increase the risk of malignant transformation [9]. These cytokines are thought to act by providing necessary microenvironment and genetic aberration(s) for lymphoma pathogenesis [10]. Host-derived cytokines can promote growth or attenuate apoptosis to facilitate development, invasion and metastasis of cancer. Unresolved host immune reactivity in form of cytokines may provide crucial environment for BL tumour development in endemic disease geographic locations. Our study aimed at describing cytokines in children and adolescents with BL at Moi Teaching and Referral Hospital (MTRH) in western Kenya.

## Main text

### Methods

Children and adolescents aged ≤ 18 years with clinical and histology diagnoses of BL or NHL at Moi Teaching and Referral Hospital (MTRH) were consecutively recruited to the study between January 2011 and December 2013, before they initiated chemotherapy. The hospital is a major health provider for cancer patients in western Kenya. Ethical clearance was obtained from Institutional Research Ethics Committee (IREC) of MTRH and Moi University before the study begun and written informed consent to participate was sought from parent or legal guardian of each participant. Potential participants with benign conditions were excluded. Participants’ hospital files and questionnaires were used to obtain demographic and clinical information. Histology and immunohistochemistry (IHC) panel of CD10, CD20, CD38, CD44, BCL-2, Ki-67, and MYC protein antibodies were used to delineate BL from non-BL conditions. Four (4) ml of blood samples were drawn from the median antecubital vein of the upper limb using EDTA BD Vacuitainer^®^ Blood Collection sets. Haematology analyzer, Coulter^®^ AcT5 Diff CP (Beckman Coulter, USA), was used to determine absolute counts (cell/µl) of white blood cells, red blood cells, platelets, haemoglobin (g/dl) and lymphocytes (%). Plasma was then separated by centrifugation at 1013 × *g* for 5 min and stored at - 80 °C in duplicate aliquots of 1000 μl until use. Cytokines IL-2, IL-4, IL-6, IL-10, TNF-α, IFN-γ, and IL-17A were simultaneously detect using BD^®^ CBA Human Th1, Th2 and Th17A kit (#560484). This kit is comprised of seven-bead populations, each population with distinct fluorescence intensity and coated with capture antibodies specific for each of the cytokines’ protein. Cytokine capture beads and recombinant standards or samples were incubated and followed by staining with phycoerythrin (PE) conjugated detection antibodies to form sandwich complexes. The intensity of PE fluorescence of each sandwich complex was related to the concentration of various cytokines. After appropriate incubation, standards and samples were acquired in a FacsCalibur^®^ flow cytometer and resulting data analysed by FCAP Array^®^ software to generate concentrations of various cytokines. Soluble human TGF-β1 in the plasma samples were determined in a single plex assay using BD CBA HumanTGF-β1 Single Plex Flex Set kit (#560429) and BD Human Soluble Master Buffer kit (#558264), following the manufacturer’s instructions. After appropriate incubation, standards and samples were acquired in a FacsCalibur^®^ flow cytometer and resulting data analysed using FCAP Array^®^ software to generate concentrations of TGF-β1 protein. Parents or guardians were later followed up within a year for information on treatment outcome of their children.

Collected data were entered into computer for storage and initial analysis. Descriptive analyses were performed by using frequency tables for categorical variables and for continuous variables, measures of central tendency were done using SAS version 9.1 (SAS Institute, Cary, NC), SPSS^®^ version 20 software. Statistical significances of differences in means were calculated by Mann–Whitney rank-sum test. All p-values reported were obtained using two-sided tests of statistical significance, p < 0.05.

### Results

33 (32%) of 104 study participants were confirmed BL and the remaining categorised as non-BL using histology and consensus IHC panel of antibodies. Of the BL participants 78.8 and 21.8% were males and females respectively, majority (75.8%) of them aged between 5 and 12 years. The age of BL participants ranged from 3 to 16 and a mean of 8.8 years (standard deviation [SD] 3.7) at diagnosis.

**Fig. 1:**
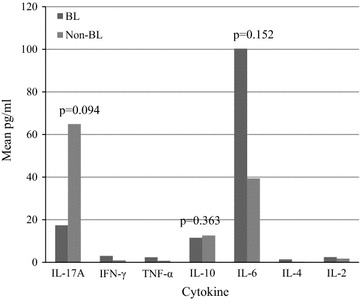
Shows expressions mean (pg/ml) of cytokine IL-17A, INF-γ, TNF-α, IL-10, IL-6, IL-4, IL-2 in Burkitt’s and non-Burkitt’s lymphoma groups

**Fig. 2:**
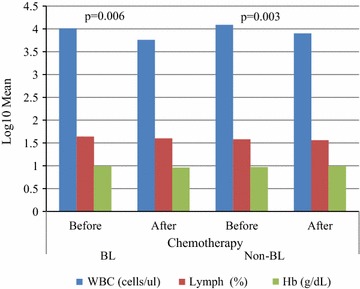
Shows log10 mean (cell/μl) of WBC in Burkitt’s and non-Burkitt’s lymphoma participants before and after appropriate chemotherapeutic regimen

The mean IL-6 levels (pg/ml) were 100.3 and 39.4 p = 0.152; IL-10 11.5 and 12.5 p = 0.363; IL-17A 17.8 and 64.9 p = 0.094 in BL and non-BL respectively (Fig. 1). In BL’s, the medians (interquartile range [IQR] pg/ml) of IL-6, in BL was 13.8 (10.1–31.1), IL-10 3.3 (0.6–7.0), IL-17A 0 (0–4.1) while in non-BL’s IL-6 9.0 (1.7–20.4), IL-10 1.5 (0.6–6.6) and IL-17A 5.2 (0–32.0). The differences in the levels of cytokines IFN-γ, TNF-α and IL-2 cytokines were not dramatic between BL and non-BL participants. White blood cell count (WBC) and platelets were slightly above median normal values. However, the log10 of mean of WBC before and after chemotherapy were significantly different in BL cases (p = 0.006) and non-BL cases (p = 0.003) (Fig. 2).

### Discussion and conclusion

Burkitt’s lymphoma occurred more (78.8%) in male children and adolescents than their female counterparts. The male to female ratio of 4:1 agreed with previous studies in Kenya [11, 12] but higher than that reported in Tanzania by Mwakigonja et al. [13]. It is not certain whether the apparent male bias was due to socio-environmental or biological factors among the study participants.

The levels of IL-6, an inflammatory cytokine, in BL participants in this study were higher, though not statistically significant, but similar in trend to Aka et al. [14]. The relatively high levels of IL-6 cytokines in BL than in non-BL participants was also comparable to earlier reports of increased levels of IL-6 and other inflammatory cytokines in lymphomas by Mellgren et al. [15], but different from Oduor et al. [16], who reported no association between IL-6 expression and BL. A study by Chopra et al. [17] demonstrated increased IL-6 levels in BL cases compared to healthy controls. Inflammatory cytokines other than IL-6 have been associated with lymphoma pathogenesis [18]. Interleukin 6 is a known potent B cell stimulatory factor and may also influence the general condition of paediatric patients leading to BL development. The observed higher levels of IL-6 in BL cases, in this study, could be attributed to the interaction between the host immune system and prevalent infections such as EBV and *P. falciparum* in study region. The main source of IL-10, an anti-inflammatory cytokine, in the groups was unclear but may also be related to persistent infections in the study region. Moormann et al. [19] found a positive correlation between IL-10 and malaria parasite densities in children with less-effective *P. falciparum* clearance in western Kenya. Endemic *P. falciparum* infection is usually biased toward anti-inflammatory cytokines such as IL-10 among others. Moreover, one of the EBV latency programme products BCRF-1, is human IL-10 homologue [20], and may have contributed to expression and levels of IL-10 observed in this study. Interleukin 10 in cell microenvironment provided a reduced cytotoxicity and potential for BL development. Augmented levels of IL-6 and IL-10 have been reported in EBER^+^ BL lines when compared to EBER^-^ BL lines by Brady et al. [21]. Bower [22], reported that simultaneous infection enhanced growth of EBV transformed lymphoblastoid cell lines in vitro. This implies that cytokines in response to prevailing may participate in the development of BL in-vivo. In this study, no distinction was made between viral and human IL-10.

The Th1 cytokines represented in this study by IFN-γ, IL-2 and TNF-α were relatively lowly expressed and did not differ significantly between the study groups, similar to previous studies by Zeigler [23]. Possibly indicating that the observed levels IL-10 inhibited Th1 cytokines in BL’s but not Th17 responses in non-BL group. The Th1 cytokines are important in immunosurveillance and control of infections including EBV, a process that requires human leukocyte antigen (HLA) class I restricted CD8^+^ cytotoxic T lymphocyte (CTL). The mechanisms by which IFN-γ deficiency promotes increased tumour formation are multifactorial including; less than optimal control of tumour-cell growth, apoptosis of T cell through Fas-FasL pathway and increased angiogenesis. Impaired NFκB signalling and genes involved B cell receptor signalling such as STAT1 and STAT2, have been found to be involved in IFN-γ down-regulation in BL cases compared to B-lymphoblastoid cell line [24]. Negative regulation of STAT1 by MYC protein, expressed in many BL cases in this study (data not shown) may have blocked STAT1 expression or indirectly suppressed IFN-γ induction in these BL participants. Thus, expression of *c*-*myc* gene, in BL’s in this study, decreased immune responses to EBV-positive B cells by altering genes in the NF-κB pathway. By so doing, MYC protein then enhance tumour cell survival and facilitate immune evasion [25]. Immune response cytokines of particular compartment and genetic aberrations occasioned by prevalent infections may underlie BL-genesis. Compared to normal ranges, WBC counts were only slightly depressed in both BL and non-BL participants, suggesting no bone marrow or leukemic involvement in the study participants. This differed from Kelemen et al. [26], who found elevated WBC levels in Burkitt’s lymphoma with atypical morphology. The levels of WBC values tended to be normal ranges after treatment, possibly due to positive response impact of chemotherapy.

Burkitt’s lymphoma children and adolescents in the study setting showed moderately higher levels of IL-6, IL-17A, IL-10 but low levels of IL-2, TNF-α and IFN-γ, possibly determined by prevailing infections. These cytokines may influence BL pathogenesis and may be potential therapeutic targets. The distinction between human and viral IL-10 homologue and relationship with BL risk need further studies.

### Limitations

All the samples were stored at - 80 °C for up to six months before analysis, we did not validate stability of different cytokines at the storage temperature and/or adherence to storage vessel used due to lack of enough reagents. The differences observed between the study groups may have been limited by the small sample size used. Loss to follow up was a challenge and it is likely that self-reported demographic characteristic may be subjective and prone to recall bias.

## Abbreviations

BD: Becton–Dickinson
CBA: cytometric bead array
c-myc: c-myelocytomatosis gene
IFN-γ: interferon γ
IL: interleukin
TGF-β: transforming growth factor β
Th: T helper cell
TNF-α: tumour necrosis factor α
